# Exploring the Mechanism of Berberine Intervention in Ulcerative Colitis from the Perspective of Inflammation and Immunity Based on Systemic Pharmacology

**DOI:** 10.1155/2021/9970240

**Published:** 2021-06-09

**Authors:** Yan Jiang, Li Zhao, Qing Chen, Lihong Zhou

**Affiliations:** ^1^School of Medicine, Hunan Normal University, Changsha, Hunan, China; ^2^The First Affiliated Hospital, University of South China, Hengyang, Hunan, China

## Abstract

**Background:**

Ulcerative colitis (UC) is a chronic nonspecific inflammatory disease of the colon and rectum. Recent studies found that berberine had effects on inflammatory diseases and immune diseases.

**Methods:**

The PharmMapper database was used to predict the berberine potential target and GeneCards database and OMIM database were utilized to collect UC genes. The Cytoscape software was used to construct and analyze the networks and DAVID was utilized to perform enrichment analysis. Then, animal experiments were performed to validate the prediction results. The experimental rats were randomly divided into normal group (control group), model group, and berberine group. The general condition, body weight, gross morphology of colon tissue, and colonic mucosal damage index (CMDI) score were observed. The pathological changes of colon tissue were observed by H&E staining. The levels of serum interleukin-1*β* (IL-1*β*), tumor necrosis factor-*α* (TNF-*α*), and IL-4 were detected by ELISA. The expressions of IL-1*β*, TNF-*α*, and IL-4 protein in colon tissue were detected by immunohistochemistry.

**Results:**

A total of 211 Berberine's potential targets and 210 UC genes were obtained. The enrichment analysis showed that berberine may regulate inflammation, inflammatory cytokines, and their mediated inflammation signal pathways such as inflammatory bowel disease (IBD), rheumatoid arthritis, cytokine-cytokine receptor interaction, TNF, T cell receptor, Toll-like receptor, and JAK/STAT signaling pathway. Compared with the model group, the body mass of rats in the berberine group was significantly increased (*P* < 0.05); the general morphology and pathological changes of colon tissue were significantly improved; CMDI score, serum and colon tissue IL-1*β*, TNF-*α* content, and protein expression were decreased significantly (*P* < 0.05); and IL-4 content and protein expression increased significantly (*P* < 0.05).

**Conclusion:**

Berberine can interfere with UC through related biological processes and signal pathways related to inflammation and immunity. In-depth exploration of the mechanism of berberine in the treatment of UC will provide a basis for clinical application.

## 1. Introduction

Inflammatory bowel disease (IBD) mainly includes ulcerative colitis (UC) and Crohn's disease (CD). The number of IBD patients in Europe and the United States has accounted for 0.5% of the world's population. At present, retrospective studies on the incidence and prevalence of IBD worldwide show that the incidence in Europe and the United States has reached a plateau, while other countries are in the first stage of the growth of new cases [1]. By 2025, IBD patients in China will reach 1.5 million [2, 3]. UC is a chronic nonspecific inflammatory disease of the colon and rectum, and the lesions are limited to the large intestine mucosa and submucosa. Current studies have shown that the pathogenesis of UC is complex, involving the combined effects of various factors such as genetic susceptibility, epithelial barrier defects, dysregulated immune responses, and environmental factors [4, 5]. The main treatments for UC include the following: (1) Sulfasalazine salicylic acid preparations, such as Mesalazine; (2) corticosteroids, such as prednisone or dexamethasone; and (3) immunosuppressants [6, 7]. However, these drugs are limited due to their limited clinical efficacy and large side effects. Meanwhile, TNF inhibitors are only effective in about half of patients [8]. At present, natural plant products, especially plant component monomers, are getting more and more attention in the treatment of UC [9, 10].

Berberine is an isoquinoline alkaloid extracted from the root and bark of the *Coptis chinensis* plant of the Ranunculaceae family [11]. Recent studies have shown that berberine has high value in the clinical application of tumors, diabetes, cardiovascular diseases, inflammatory diseases, and immune diseases. Its main mechanism is anti-inflammatory immune response and antioxidant [12–14]. Studies have also shown that berberine can prevent the production of proinflammatory cytokines in colitis [15–19]. The mechanism may be that berberine inhibited lipopolysaccharide- (LPS-) induced cytokine production and the activation of MAPK and NF-*κ*B in macrophages [17]. Meanwhile, berberine regulates the balance of Treg/Th17 to treat UC by regulating the intestinal flora in the colon [20]. Berberine also exerts a protective effect on the colon of UC by regulating the interaction of enteric glial cells-intestinal epithelial cells-immune cells [21]. Berberine can also interfere with mucosal inflammation driven by oncostatin M to treat chronic UC [22]. However, the network biological mechanism of berberine in the treatment of UC is still unknown.

Systemic pharmacology, as a product of multidisciplinary intersection, includes multiple research methods such as classical pharmacology, chemical biology, and bioinformatics and covers a large number of experimental disciplines including research techniques from cells and tissues to organs [23, 24]. Systemic pharmacology studies the occurrence and development of diseases from the perspective of biological networks, understands the interaction between drugs and the body, and guides the discovery of new drugs [25]. Therefore, this study hopes to explore the molecular mechanism of berberine intervention in the UC disease network through systemic pharmacological strategies.

## 2. Materials and Methods

### 2.1. Berberine's Potential Target Prediction and UC Genes Collection

PubChem Compound database search was used to retrieve the PubChem CID of berberine. Then PubChem CID was imported into Open Babel software to obtain the 3D structure of the compound and saved as a mol2 format file. Then, the mol2 format file of berberine was uploaded to the PharmMapper online database (http://lilab-ecust.cn/pharmmapper/) [26], and the reverse pharmacophore method was used for target prediction.

“Ulcerative colitis” was used as a key word, and the OMIM (http://omim.org/) database [27] and GeneCards (http://www.genecards.org) [28] were searched to obtain UC-related genes. In GeneCards, the genes with relevance score > 12.0 were selected for sequence research. The UniProt database is used to correct all target names into Official Symbol (Table S1 and Table S2).

### 2.2. Network Analysis and Enrichment Analysis Methods

The berberine targets and UC genes were input into String (http://string-db.org/) to obtain the protein-protein interaction (PPI) data [29]. The Cytoscape software was utilized to construct and analyze the networks [30]. The nodes in the network represent targets, genes, and so forth, and edges show the relationship among nodes. The “Network Analyzer” plug-in was used to analyze the network topology. The “MCODE” plug-in was utilized to detect the closely connected part of PPI network.

Targets and genes were imported into DAVID (https://david.ncifcrf.gov/), and the species was defined as “human” for gene ontology (GO) enrichment analysis and Kyoto Encyclopedia of Genes and Genomes (KEGG) pathway enrichment analysis [31].

### 2.3. Experimental Materials

#### 2.3.1. Experimental Animal

Sixty specific-pathogen-free- (SPF-) grade SD rats, male and female, weighing 220 + 10 g, were purchased from Changsha Tianqin Biotechnology Co., Ltd., animal license number SCXK (Xiang), 2018–0002. SD rats were raised in the animal room of the First Affiliated Hospital of University of South China, with a temperature of 26 ± 10°C and a relative humidity of 50% ± 10%. The rats were adaptively fed for one week. The procedures for care and use of animals were approved by the Ethics Committee of the First Affiliated Hospital of University of South China (USCAF-047) and all applicable institutional and governmental regulations concerning the ethical use of animals were followed.

#### 2.3.2. Experimental Drugs, Reagents, and Instruments

The experimental drugs, reagents, and instruments are as follows: berberine hydrochloride (Shanghai Xinyi Tianping Pharmaceutical Co., Ltd., National Medicine Standard H31021444, 0.1 g/tablet), dextran sulfate sodium salt (DSS) (Sigma Inc., batch number 31404-5G-F), H&E staining kit (Wuhan Boster Biological Technology Co., Ltd., batch number AR1180), interleukin-1*β* (IL-1*β*), tumor necrosis factor-*α* (TNF-*α*), IL-4 ELISA kit (Ambion company, catalog numbers xyR013, xyR017, and xyR097), IL-8 ELISA kit (Nanjing Senbega Biotechnology Co., Ltd., batch number: SBJ-R0700), Rat IL-6 ELISA kit (Cat. No. EK0412), Rat IL-12 (P70) ELISA kit (Cat. No. EK1652), Rat IL-10 ELISA kit (Cat. No. EK0418) (Wuhan Boster Bioengineering Co., Ltd.), rabbit anti-mouse IL-1*β*, TNF-*α*, IL-4 antibodies (Beijing Boaosen Biotechnology Co., Ltd., batch numbers bs-10859R, bs-10802R, and bs-1740R), horseradish enzyme-labeled goat anti-rabbit IgG secondary antibody (Beijing Zhongshan Jinqiao Biotechnology Co., Ltd., batch number ZB1208), rotary microtome (Leica RM2235 rotary microtome), optical scanning microscope (Zeiss Optical Instruments Beijing Branch), and microplate reader (Rui Xuan Electronic Technology Co., Ltd.).

### 2.4. Experimental Methods

#### 2.4.1. Animal Modeling

According to [32], 40 rats were randomly selected and fed with a normal diet for 7 days. On the 8th day, they were given distilled water supplemented with 2% DSS for 7 days and then given only distilled water for 14 days. The above process is carried out in 3 cycles, and the last two cycles are 1.5% DSS/distilled water for 5 days + distilled water for 6 days. Rats with reduced foraging, decreased body weight, loose stools, mucus pus, and blood in the stool represented the successful modeling. The remaining 20 rats were given an equal volume of normal saline instead of DSS as normal group. The changes in diet, body mass, and stool traits of experimental animals were observed and recorded.

#### 2.4.2. Animal Grouping and Intervention

The successfully modeled rats were randomly divided into 2 groups (model group and berberine group) according to the random number method, with 20 rats in each group, half males and half females. The 20 rats that were not modeled represented the normal group. The berberine group was given berberine 1.8 g/kg by gavage 7 days after the model was established. The normal group and the model group were given an equal volume of normal saline. The intervention was performed once a day for 17 consecutive days.

#### 2.4.3. Specimen Collection

After fasting for 24 hours after the last administration, the rats were anesthetized with 2% sodium pentobarbital 0.3 ml/100 g. After blood collection, the rats were sacrificed by cervical dislocation; colon tissues were collected for index detection and stored at −20°C.

#### 2.4.4. General Morphological Observation of Rat Colon

The colon was cut along the longitudinal axis and rinsed with normal saline. According to Luketal's standard [33], the degree of colonic mucosal damage was scored. 0 points indicate no inflammation and ulcers. 1 point indicates local hyperemia but no ulcers. 2 points indicate hyperemia and thickening of the intestinal wall but no ulcers. 3 points indicate 1 ulcer and inflammation, about 0 to 1 cm in diameter. 4 points indicate 2 or more ulcers and inflammations, about 1.1 to 2 cm in diameter, but there is no adhesion between the intestine and peripheral organs. 5 points indicate that the ulcer extends more than 2 cm, the intestine is thickened, and the adhesion to the surrounding organs is serious.

#### 2.4.5. Pathological Observation of Rat Colon

The colon tissue was fixed with 4% paraformaldehyde, decalcified, dehydrated, permeabilized, and embedded in paraffin for H&E staining. The histological changes were observed under the microscope.

#### 2.4.6. Serum IL-1*β*, TNF-*α*, IL-4, IL-6, IL-8, IL-10, and IL-12/P70 Contents Detection

The IL-1*β*, TNF-*α*, IL-4, IL-6, IL-8, IL-10, and IL-12/P70 contents were detected according to the instructions of the ELISA kit. Rat IL-1*β*, TNF-*α*, IL-4, IL-6, IL-8, IL-10, and IL-12/P70 monoclonal antibodies were coated on the ELISA plate, and the corresponding proteins in the standards and samples are monoclonal antibody binding. Then the corresponding biotinylated antibody was added to form an immune complex. Then horseradish peroxidase-labeled streptavidin was combined with biotin, and the enzyme substrate OPD was added. After the yellow color appeared, the stop solution was added. The absorbance (A) was measured at 450 nm wavelength of the microplate reader, and the contents of IL-1*β*, TNF-*α*, IL-4, IL-6, IL-8, IL-10, and IL-12/P70 were calculated according to the A value of the sample to be tested.

#### 2.4.7. Detection of IL-1*β*, TNF-*α*, and IL-4 Protein Expression in Colon Tissue

1 cm^3^ rat colon tissue was fixed in 4% paraformaldehyde for 30–60 min, washed twice with PBS, dehydrated at 50°C, embedded in paraffin, and sectioned (about 4 *μ*m). All samples are deparaffinized, hydrated, inactivated, and processed for antigen retrieval. After the sections were stained by immunohistochemistry, they were observed under a microscope. The Motic Advanced 6.0 image analysis system was used for analysis.

### 2.5. Statistical Analysis

SPSS19.0 statistical software was used for statistical analysis. The measurement data is expressed as mean ± SD (x¯ ± s). The samples were first tested for normality and homogeneity of variance. If the variance was uniform, the LSD method was used for multiple comparisons between groups; if the variance was not uniform, the nonparametric rank-sum test was used for comparison, and the Kruskal-Wallis H test was used to compare the total difference.

## 3. Results

### 3.1. Berberine's Potential Target Prediction and UC Genes

**Figure 1:**
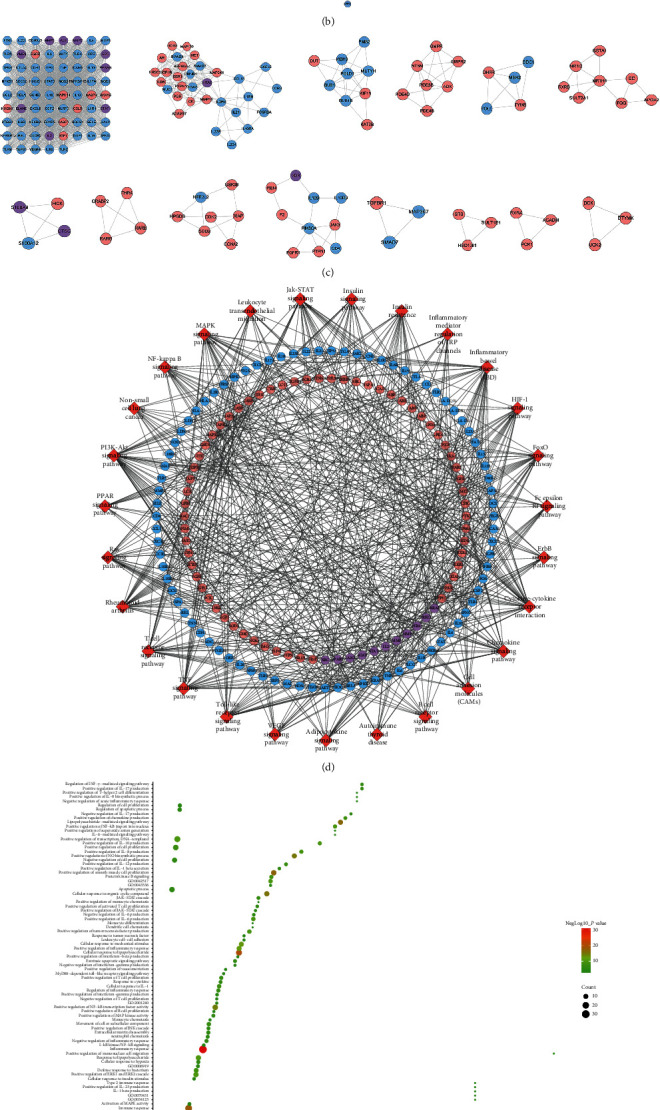
Network analysis results. (a) Venn diagram of berberine's potential targets and UC genes. (b) Berberine-UC PPI network. (c) Clusters of berberine-UC PPI network. (d) Signaling pathway of berberine-UC PPI network. (e) Bubble chart of biological processes in cluster 1. (f) Bubble chart of signaling pathway. Red diamonds stand for signaling pathways; pink circles stand for berberine potential targets; blue circles stand for UC genes; purple circles stand for berberine-UC target. *x*-axis stands for fold enrichment.

A total of 211 berberine's potential targets and 210 UC genes were obtained. There are some intersections between the berberine target set and the UC gene set (Figure 1(a)). The targets shared by the two target sets are MMP9, HRAS, STAT1, PRKCQ, MMP2, VDR, S100A9, IL2, PLA2G2A, ELANE, CTSG, PPARG, MMP3, AURKA, SRC, ALB, and BRAF.

### 3.2. Berberine-UC PPI Network Analysis

#### 3.2.1. Berberine-UC PPI Network

This network is composed of 191 berberine potential target nodes, 151 UC gene nodes, 17 berberine-UC target nodes, and 6742 edges. The top 20 targets (according to the degree) can be divided into three categories: (1) berberine target set: EGFR (140 edges), CASP3 (128 edges), and MAPK14 (113 edges); (2) UC gene set: IL-6 (185 edges), TNF (181 edges), AKT1 (173 edges), TP53 (163 edges), VEGFA (157 edges), STAT3 (146 edges), IL-10 (139 edges), TLR4 (133 edges), CXCL8 (128 edges), IL-1*β* (125 edges), PTGS2 (118 edges), and IL-4 (114 edges); and (3) berberine-UC target set: ALB (169 edges), MMP9 (126 edges), SRC (123 edges), IL-2 (120 edges), and HRAS (111 edges) (Figure 1(b)).

#### 3.2.2. Biological Processes of Berberine-UC PPI Network

The berberine-UC PPI network was analyzed by MCODE and returns 14 clusters (Figure 1(c)). The berberine targets and UC genes in those clusters were input into DAVID to perform GO enrichment analysis.

Cluster 1 is mainly related to inflammation (inflammatory cells, e.g., macrophages, neutrophil proliferation, etc.), inflammatory cytokines (IL-10, IL-6, IL-8, IL-1*β*, IL-12, and IL-17), and their mediated inflammation signal pathways (NF-*κ*B, JAK/STAT, and Toll signaling pathway); immune response (immune cells, e.g., TH1 and TH17 cells activate and proliferate, B cells activate and proliferate) and immune cytokines (various chemokines and INF-*α*, INF-*β*, INF-*γ* and their mediated immune response); and apoptosis and proliferation. Cluster 2 is mainly related to immune response (such as positive regulation of interleukin-17, T-helper type 17 cellular immune response activation, T cell proliferation, T-helper type 1 immune response activation, memory T cell differentiation, and chemokine-mediated signaling pathway). Cluster 4 is mainly related to purine nucleotide metabolism. Cluster 5 is mainly related to mitosis and tetrahydrofolate metabolism. Cluster 6 is mainly related to the metabolism of steroid hormones and their signal transduction. Cluster 8 is mainly related to steroid-mediated signaling pathways. Cluster 9 is mainly related to oxidative stress. Cluster 10 is mainly related to T cell proliferation and signaling pathways mediated by immune cytokines. Cluster 11 is mainly related to the transforming growth factor pathway. Clusters 3, 7, 12, and 14 did not return any UC-related biological processes. Cluster 13 failed to return any human biological processes (Table S3). The biological processes in cluster 1 were shown in Figure 1(e) as an example.

#### 3.2.3. Signaling Pathways of Berberine-UC PPI Network

The targets and genes in berberine-UC PPI network were input into DAVID to perform pathway enrichment analysis and return sixteen signaling pathways (Figure 1(d)). The top 10 signaling pathways are as follows: inflammatory bowel disease (IBD), rheumatoid arthritis, TNF signaling pathway, T cell receptor signaling pathway, cytokine-cytokine receptor interaction, Toll-like receptor signaling pathway, JAK/STAT signaling pathway, FoxO signaling pathway, HIF-1 signaling pathway, and insulin resistance (Figure 1(f)). The details of the signaling pathway were shown in Table S4.

### 3.3. General Condition of Rats

**Figure 2:**
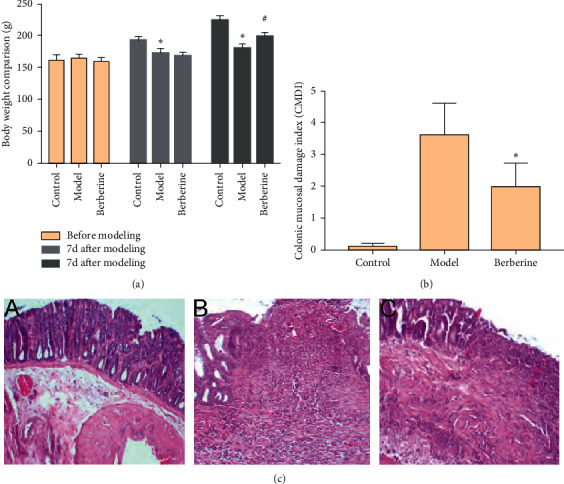
(a) The body weight. (b) Effect of berberine on CMDI. (c) Pathological changes of colon (compared with normal control group, ^*∗*^*P* < 0.05 and ^*∗∗*^*P* < 0.01; compared with model group, ^#^*P* < 0.0 and ^##^*P* < 0.01; H&E staining, x100; A, normal group; B, model group; and C, berberine group).

One day after the establishment of the model, the rats were depressed, with dull and curly hair and reduced activity, and accompanied by mucus stools, bloody stools, and decreased body weight. Compared with the normal control group, the body weight of the model group was significantly reduced, and the difference was statistically significant (*P* < 0.01). Compared with the model group, the body mass of the berberine group increased significantly, and the difference was statistically significant (*P* < 0.01) (Figure 2(a)).

### 3.4. Effect of Berberine on Colonic Mucosa Damage Index (CMDI)

Compared with the normal group, the CMDI score of the model group was significantly increased (*P* < 0.05). Compared with the model group, the CMDI score of rats in the berberine group was significantly decreased (*P* < 0.05) (Figure 2(b)).

### 3.5. Pathological Changes of Colon

There was no or rare inflammatory cell infiltration in the colon tissue of rats in the normal group. In the model group, the mucosa of the colon tissue of rats was obviously hyperemia, edema, extensive inflammatory cell infiltration, and decreased goblet cells; the glands around the ulcer are defective and disorderly distributed, and the intestinal wall is extensively fibrotic; the lesion reaches deep into the submucosa, muscle layer, and even serosal layer; and inflammatory granuloma can be seen. Compared with the model group, the pathological changes in the colon of the berberine group were significantly improved; its manifestations as mild hyperemia, edema, reduced inflammatory cell infiltration, and shallower inflammatory cell infiltration, mainly concentrated in the mucosal layer and submucosa, with a small amount of fibrosis in the intestinal wall; new glands can be seen around some colon ulcers, and new goblet cells and intestinal mucosa can be seen in the intestinal wall (Figure 2(c)).

### 3.6. Effect of Berberine on Serum IL-1*β*, TNF-*α*, and IL-4 Levels

**Figure 3:**
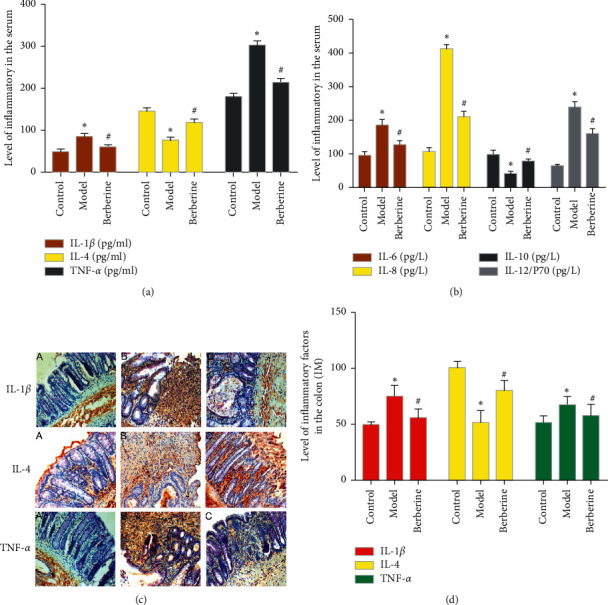
(a) Effect of berberine on serum IL-1, TNF-*α*, and IL-4 levels. (b) Effect of berberine on serum IL-6, IL-8, IL-10, and IL-12/P70 levels. (c) IL-1, TNF-*α*, and IL-4 in colon tissue (immunohistochemistry, x200). (d) Expressions of IL-1, TNF-*α*, and IL-4 in colon tissue (^∗^compared with normal control group, *P* < 0.05; ^#^compared with model group, *P* < 0.05; A: normal group; B: model group; and C: berberine group).

Compared with the normal group, the serum levels of IL-1*β* and TNF-*α* in the model group increased significantly, and the levels of IL-4 decreased significantly (*P* < 0.05). Compared with the model group, the levels of serum IL-1*β* and TNF-*α* in the berberine group were significantly reduced, and the levels of IL-4 were significantly increased (*P* < 0.05) (Figure 3(a)).

### 3.7. Effect of Berberine on Serum IL-6, IL-8, IL-10, and IL-12/P70 Levels

Compared with the normal group, the cytokines IL-6, IL-8, and IL-12 were significantly increased after modeling (*P* < 0.05), while IL-10 was significantly decreased (*P* < 0.05). The expression and secretion of these cytokines are positively correlated with the degree of inflammation of the disease, and they also show that the modeling is successful. After intervention, compared with the model group, IL-6, IL-8, and IL-12 in the berberine group were significantly reduced (*P* < 0.05), and IL-10 was significantly increased (*P* < 0.05). This shows that the cytokine changes after treatment and the treatment is effective (Figure 3(b)).

### 3.8. Effect of Berberine on Colon Tissue IL-1*β*, TNF-*α*, and IL-4 Levels

Compared with the normal group, the expressions of IL-1*β* and TNF-*α* protein in the colon tissue of the model group increased significantly, and the expression of IL-4 protein decreased significantly (*P* < 0.05). Compared with the model group, the expressions of IL-1*β* and TNF-*α* protein in the colon tissue of the berberine group were significantly reduced, and the expression of IL-4 protein was significantly increased (*P* < 0.05) (Figures 3(c) and 3(d)).

## 4. Discussion

TNF-*α*, IL-6, IL-1*β*, and COX2 are the most important targets for UC. Berberine may indirectly act on those disease targets by acting on multiple targets such as SRC, CASP3, MAPK8, and JAK2. The levels of TNF-*α*, IL-6, and IL-1*β* reflect the disease process. Clinically, the international guidelines for the medical management of UC recommend the use of anti-TNF monoclonal antibodies and vedolizumab for the treatment of UC [34].

GO enrichment analysis showed that berberine is mainly involved in biological processes and molecular functions such as cytokine regulation, cell survival, migration, proliferation, and apoptosis. KEGG enrichment analysis also showed that these targets are mainly enriched in pathways related to inflammation, immunity, and tumors, such as JAK/STAT3, NF-*κ*B, and TNF signaling pathways, which are related to the development of ulcerative colitis. NF-*κ*B and STAT3 mainly exist in the cytoplasm. When they are subjected to appropriate external stimuli such as TNF-*α*, IL-6, and LPS, NF-*κ*B and STAT3 are activated. The activated NF-*κ*B and STAT3 enter the nucleus and bind with DNA to mediate the expression of downstream inflammatory factors (TNF-*α*, IL-1*β*, and COX2) [35]. Current research shows that UC is mainly related to the immune and intestinal flora caused by multiple signaling pathways such as PI3K/AKT/mTOR, NF-*κ*B, TLR4/My D88/NF-*κ*B, MAPK, JAK/STAT, Wnt, and Notch signaling pathway [36, 37]. The current phase I clinical study shows that berberine can significantly reduce the Geboes grade of colon tissue [38]. The mechanism may be related to the inhibition of Th17 and the regulation of the interaction of enteric glial cells-intestinal epithelial cells-immune cells [21, 39]. Berberine mainly relieves colitis by improving the intestinal barrier function and promoting anti-inflammatory and antioxidative stress responses. Its anti-inflammatory mechanism may be related to blocking the IL-6/STAT3/NF-*κ*B signaling pathway [40–42].

In recent years, the incidence of UC in our country has been increasing year by year, and it has become a common clinical refractory disease. The pathogenesis of UC is mainly related to heredity, immunity, microorganisms, and so forth; immune factors occupy an important position among them [43]. The mechanism is that abnormal intestinal mucosal immune system leads to infiltration of a large number of inflammatory cells in the intestine. The massive release of inflammatory factors causes the necrosis and shedding of intestinal epithelial cells and reduces the intestinal mucosal barrier function [43, 44]. SIgA is an important immunomodulatory protein in the intestinal tract. It acts as the first line of defense to maintain the homeostasis of the intestinal mucosa by protecting the integrity of the intestinal biological barrier and regulating the immune response [45]. In the course of immune response, the activation and proliferation of colonic lamina propria (LP) T lymphocytes are extremely important for maintaining intestinal immune homeostasis [45, 46]. In addition, the imbalance of proinflammatory cytokines/anti-inflammatory cytokines is an important reason for the disorder of intestinal mucosal immune function [47]. The role of proinflammatory factor IL-I*β* and anti-inflammatory factor IL-4 has become increasingly prominent. In particular, IL-1*β* causes the production of inflammatory transmitters such as TNF-*α*, IL-6, and IL-8 through autocrine or paracrine methods, causing acute inflammation in local tissues, which in turn causes colonic tissue congestion, edema, ulcers, necrosis, and so forth [48]. IL-4 can not only inhibit the occurrence and development of inflammation but also has a certain immunosuppressive function, so it is very important in maintaining the stability of the intestinal mucosal environment [49]. The serum levels of IL-1*β*, IL-4, and TNF-*α* in UC patients can be used as important indicators for evaluating the condition and prognosis of UC patients. This study found that, after the UC model was established, the general condition and body weight of the rats in the model group were significantly reduced, the CMDI score was significantly increased, and the colon tissue showed different degrees of pathological damage, which was consistent with the changes in UC. Berberine can improve the general condition and body weight of UC rats to varying degrees, reduce the CMDI score, and improve the gross morphology and pathological damage of the colon, suggesting that berberine can significantly improve the general morphological changes and pathological damage of UC rats.

Meanwhile, after modeling, the serum and colon tissue IL-1*β* and TNF-*α* levels increased, and the IL-4 level decreased. After the intervention of berberine, the levels of IL-1 and TNF-*α* and protein expression in rat serum and colon tissue were significantly reduced, and the IL-4 levels were significantly increased. This suggests that the improvement of berberine's immune function may be related to the regulation of the protein expression of cytokines such as IL-I*β*, TNF-*α*, and IL-4.

## 5. Conclusion

In summary, berberine can interfere with UC through related biological processes and signaling pathways related to inflammation and immunity. Berberine can significantly improve the general morphology and pathological changes of the colon tissue of UC rats induced by DSS, and it can regulate the immune function of the body by affecting the expression levels of inflammatory cytokines and intestinal mucosal immune proteins. In the future, we will deeply explore the mechanism of berberine in the treatment of UC so as to provide a basis for clinical application.

## Data Availability

The data used to support the findings of this study are included in the article and the supplementary information files.

## Supplementary Materials

Supplementary Materials Table S1: berberine potential targets. Table S2: UC genes. Table S3: biological processes of clusters. Table S4: signaling pathways.
