# Correction to Exploring the Mechanism of Berberine Intervention in Ulcerative Colitis From the Perspective of Inflammation and Immunity Based on Systemic Pharmacology

**DOI:** 10.1155/ecam/9803142

**Published:** 2025-03-26

**Authors:** Yan Jiang, Li Zhao, Qing Chen, Lihong Zhou

**Affiliations:** ^1^School of Medicine, Hunan Normal University, Changsha, Hunan, China; ^2^The First Affiliated Hospital, University of South China, Hengyang, Hunan, China

Y. Jiang, L. Zhao, Q. Chen, and L. Zhou, “Exploring the Mechanism of Berberine Intervention in Ulcerative Colitis From the Perspective of Inflammation and Immunity Based on Systemic Pharmacology,” Evidence-Based Complementary and Alternative Medicine, vol. 2021, (2021). https://doi.org/10.1155/2021/9970240

In the article titled “Exploring the Mechanism of Berberine Intervention in Ulcerative Colitis From the Perspective of Inflammation and Immunity Based on Systemic Pharmacology,” there was an error in Section 2.4.2 related to the concentration of berberine administered to the animals during the experiment, where it was denoted as “1.8 g/kg.” The correct concentration should be “30 mg/kg.”

We apologize for this error.

